# How the COVID-19 pandemic is changing clinical trial conduct and driving innovation in bioanalysis

**DOI:** 10.4155/bio-2021-0107

**Published:** 2021-07-19

**Authors:** Melanie Anderson

**Affiliations:** ^1^Pharmacokinetics, Pharmacodynamics & Drug Metabolism, Merck & Co., Inc., West Point, PA 19486, USA

**Keywords:** at-home sampling, clinical trial model, COVID-19, patient centric

## Abstract

Thousands of clinical trials all over the world were stopped, disrupted or delayed while countries grappled to contain the pandemic and research resources were redeployed. The long-term effects of the turbulence caused by the pandemic have yet to be fully understood, but it should already be clear that the increased focus on participant needs and on the logistical challenges of current models are not likely to fade away quickly. This disruption is opening doors for rethinking traditional approaches to clinical trial conduct – including decentralizing site visits, introducing new methods of sample collection, rethinking matrix selection, reducing sample volumes and collaborating on device development. These approaches reduce participant burden while improving critical trial data.

## Clinical trial disruption & opportunity

The clinical trial is the cornerstone of modern pharmaceutical drug development. However, the fundamental premise in clinical research of testing the safety and efficacy of therapeutics under controlled conditions has been significantly impacted by the pandemic’s health concerns about congregate settings and ongoing social distancing requirements. In the spring of 2020, public health measures all over the globe (including mandatory lockdowns, institutional guidelines and individual behavior patterns) combined to bring clinical trials virtually to a halt [1–5]. As of January 2021 there were more than 2000 clinical trials that had been stopped explicitly due to COVID-19 restrictions [6]. Even for trials that continued, missed clinical visits and limited access to medical facilities became a universal reality as the ability to access medical facilities ended and travel restrictions were implemented. Ongoing trial conduct was meaningfully impacted, with clinical teams issuing protocol deviations as participant safety or data integrity was affected. These protocol changes needed additional Institutional Review Board approval and incurred significant additional effort and delay. In addition, new trial initiation was often delayed as enrollment of new trial participants was interrupted or halted in many trials. Concerns around treatment access, safety monitoring and trial progression became widespread in the pharmaceutical industry as the safety and protection of trial participants as well as research staff took on new emphasis [2,3,7]. In the midst of all the unknowns in a global pandemic, these safety decisions were often incredibly complicated. Many clinical trial populations still desperately needed treatment options; however, these same populations were often also be at higher risk for serious complications from COVID-19 infection [4,7].

Trial interruptions and delays have many other downstream effects. Delays can impact supply due to drug expiration. Many manufacturing activities were interrupted by pandemic prevention measures, causing further disruption. Biological samples at clinical sites or in transit experienced delays in shipping and issues during transit. Cancellations and schedule reductions in domestic and international flights further complicated sample shipments. These transit delays continued for months. Testing laboratories were also operating with diminished workforces. Entirely new ways of working have been developed as organizations moved to virtual platforms [4]. The operation of the bioanalytical laboratory was disrupted. Testing needs increased due to the pandemic but social distancing requirements placed additional burden on strained resources.

Health authorities responded to the upheaval in clinical research by providing emergency guidance on trial participant safety and ensuring compliance/quality in clinical trials [8,9]. These were intended to aid trial sponsors during these uncertain times and provide high level guidance on trial conduct. However, the challenges sponsors face associated with missing clinical site visits and corresponding missing data in clinical trials were not specifically addressed.

At the same time, organizations quickly shifted resources and efforts to research and testing of COVID-19 therapeutics and vaccines [5,10]. In this moment of global crisis, scientific and governmental and health communities worked together with unprecedented focus and collaboration, putting ‘all hands on deck’ and sharing learnings across countries, companies and institutions as never before. Within months of the outbreak, the global community understood that a new respiratory illness was emerging caused by a novel coronavirus, had mapped its genetic sequence and identified the importance of the angiotensin-converting enyzme receptor [11–13]. By April 2020, over 200 new COVID-19 clinical trials were launched resulting in both treatment and prevention solutions. These trials involved cooperation across companies, institutions and countries and have resulted in successful approaches for combating COVID-19 [14]. These include bamlanivimab, casirivimab, dexamethasone, remdesivir or tocilizumab currently recommended for treatment of COVID-19 depending on disease severity [15]. The speed of therapeutic and vaccine development has been truly amazing given the challenges associated with running clinical trials during the pandemic and can provide a roadmap for new ways of conducting trials and collaborating across stakeholders that would not have been readily imaginable in the past.

## Modernizing clinical trial conduct

The pandemic highlighted the extent to which our current site-based clinical trial model is costly, limits populations served and burdens trial participants [16–18]. In one report focusing on large, global clinical trials, around 11% of sites failed to enroll a patient, 40% of clinical site failed to meet recruitment goals and 49% of those enrolled dropped out prior to the completion of the study [19]. Even prior to the pandemic, there was a lot of interest in digital health and decentralized clinical trials [17,20,21]. The technology to enable virtually conducted clinical trials has been available for years. However, administrative barriers and traditional reimbursement cost structures have made implementation difficult [17]. But during the last year, sponsors were forced to build capabilities and processes for handling clinical trials without clinical site visits [18]. Although telehealth capabilities have existed for years, the use of telehealth expanded exponentially during 2020 in clinical research and in healthcare more broadly [20–22]. This change was due in part to the US Coronavirus Aid, Relief and Economic Security Act, which improved provider reimbursement for virtual visits [23]. Telehealth visits have become normalized for sponsors, investigators and trial participants during the pandemic. This shift in approach should have a huge impact on clinical research. I expect to see sponsors leverage this ability in the coming years to move trials from a site centric to a decentralized patient centric clinical trial model, replacing a significant portion of traditional clinical site visits with virtual tools. This shift can improve both trial efficiency and reduce patient burden [17].

There is also increasing evidence that virtual recruitment is significantly faster than traditional site-based approaches [18,24]. In one trial, recruitment was 20-times faster when implementing a ‘virtual site’ for enrollment. In addition, time for trial start up can be much lower in a decentralized design, with one Sanofi trial reporting 66% less time in study coordination when decentralized approaches were used [24]. Strategically implementing technology can improve efficiency and decrease costs associated with onboarding sites and recruitment delays.

In addition, the current clinical trial paradigm only provides access to a subset of the population. Participants need to be geographically co-located with clinical sites which can limit participation to urban centers in developed countries. Multiple reports suggest that long physical distances to clinical sites can be a significant barrier to trial participants [25–27]. This limits patient access to important therapeutics, increases enrollment timelines for a study, presents issues with trial diversity and limits deployment in rural or global settings [24]. Decentralized approaches can enable participation in trials regardless of location and can reduce the burden associated with travel and time for clinical visits [24].

Last but certainly not least, improving overall efficiency in drug development is imperative for the pharmaceutical industry. Costs keep increasing without corresponding increases in productivity [28]. Clinical trial budgets represent a significant part of the overall R&D budget in the pharmaceutical industry, and there is huge return on investment potential in using technology to reduce cost and improve efficiency in clinical research. Unproductive expenses in trial execution due to site initiations, enrollment failures and early discontinuation are costly to clinical trial budgets. In addition, late phase trials are largely conducted without understanding adherence, which can complicate assessments of safety and efficacy. In many ways, we are not getting the ‘right’ data out of trials because we do not really know if participants are actually taking their medication and what biological events are occurring between clinical site visits. Digital adherence technology is an area of increasing attention as sponsors want reliable data around dosing. Implementation of remote sampling can provide biological data outside the temporal constraints of the site visit.

There are many aspects of clinical trial conduct where decentralized or virtual methods can be employed. Generally, sponsors and clinical sites are developing hybrid models with some combination of innovative virtual techniques and traditional methods. In study initiation, using mobile technology for recruitment and informed consent forms is becoming commonplace [19]. During the conduct of the trial, telehealth visits combined with wearable devices can provide critical clinical assessments in a remote setting, eliminating the need for additional site visits. In addition, many sponsors have implemented mobile nursing to conduct these evaluations. Dosing adherence technology can be utilized to better understand compliance and the critical time of dose data point. Increasing the reliability in the time of dose data will help sponsors deconvolute safety and efficacy from adherence issues. Incorporation of new technology in clinical research is experiencing exponential growth. Recruitment and enrollment can be made more equitable as mobile approaches are able to cast a wider net. Future clinical trials will be less site centric with a shift to the home as the central site of care. Participants will be able to engage in clinical research without burdensome clinical site visits. Can we finally use technology to build clinical trial systems around the patient rather than demanding patients fit into antiquated systems built before the digital age? Digital technology has revolutionized every area of our life. Healthcare is a perennial slow adopter. The pandemic may have provided the catalyst that changes how we approach technology in our clinical trials and healthcare more broadly [29,30].

## Patient centric sampling during the pandemic

Another key element in interventional clinical trial is the collection of a biological specimen. Blood is collected for safety monitoring, biomarker evaluation, pharmacokinetics and pharmacodynamics. The physical return to the clinical site cannot be avoided unless biological samples can be obtained remotely. This sampling piece is often neglected in discussions of decentralized trials and warrants thoughtful attention. The industry must establish patient-centric sampling workflows in clinical research to enable innovation in the traditional clinical trial model. Currently, two techniques can be employed for remote collection of biological samples. The first is remote collection of a sample by a trained professional through either a phlebotomist or nurse (usually occurring in the participant’s home). The second is the implementation of new devices for remote collection by the trial participant themselves or existing care giver already present. There are advantages and disadvantages to either approach and both may have an impact on downstream analytical work.

During the early months of the pandemic, sponsors were not able to collect critical biological specimens for safety, pharmacokinetics and pharmacodynamics. In many cases, sponsors implemented alternative sampling for trials already in flight [31]. Pharmaceutical companies implemented remote nursing or mobile phlebotomy visits to conduct safety assessments and collect critical biological specimens. In most cases, traditional venipuncture approaches were still utilized, but the collection location was shifted from the clinical site to trial participants’ homes. This mitigated COVID-19 exposure risk associated with time at centralized clinical site locations. Clinical operations teams needed to quickly establish relationships with mobile phlebotomy/remote nursing vendors and develop new processes for implementation with clinical sites all over the globe. This necessity greatly expanded the logistical ecosystem of remote sampling, while maintaining the continued requirements of trial participant privacy, data collection integrity, sample quality and chain of custody.

To enable remote specimen collection, bioanalytical scientists conducted additional validation experiments to mitigate risk in this new collection paradigm. Potential sample quality issues needed to be understood. Bioanalytical laboratories determined appropriate conditions for plasma and serum isolation during remote collection, as there are limitations for temperature and centrifugation conditions in the field. In addition, storage conditions may need adjustment in remote sampling paradigms. Stability during collection and subsequent storage also needed to be established. These extra validation efforts occurred mid-study often with tight timelines and limited laboratory staff due to social distancing restrictions.

Apart from those early adaptions to existing processes, the pandemic has created a sampling revolution in terms of patient experience and expectations. COVID-19 antigen and molecular testing has become widely available both in the home and in convenient drive-through locations [32]. Simple self-sampling approaches that involve swabbing the nose or spitting in a tube have become commonplace. Individuals, employers, schools and government entities at all levels are conducting testing independently to enable the safe return to normal life – work, school, vacations and family gatherings.

In the UK, routine at home antigen testing is conducted weekly for students to attend school in person. Over the counter kits are now available in most US pharmacies. COVID-19 testing results are often available within hours available directly to the individual. This demonstrates the potential for remote sampling to provide critical data to a population, individual or healthcare provider. Furthermore, this indicates that technology is sufficiently mature to implement remote sampling approaches on a global scale. As of June 2021, 28 antigen based diagnostic tests for COVID-19 have been authorized for emergency use by the US FDA. Six of these authorized antigen tests can be conducted in the home setting [33]. Molecular diagnostic testing has over 200 authorized tests in the US. In almost all of this testing, sample collection occurs outside the traditional setting using noninvasive, painless techniques [34]. The individual participant only sees the convenience of more patient centric sampling, but behind these approaches tremendous effort has gone into developing assays, creating reagents, establishing testing laboratories and manufacturing supplies. The global analytical community responded quickly to the need for diagnostic testing and built corresponding testing infrastructure. This indicates that analytical technology is sufficiently mature to generate reliable data from samples collected remotely in drive by facilities or in the home [35]. Furthermore, remote sample collection approaches have evolved to enable simple, self collection of biological samples.

## Remote collection

Patient centric sampling with remote collection options enabled by new collection devices and matrices with optimized assays can provide critical data in clinical research. The ability to collect samples outside a clinical site visit has the potential to transform clinical trials and healthcare more broadly [31,35,36].

Biological events do not always coincide with the clinical site visit. Can we collect samples outside the clinical site visit that correspond with important biological events? In the present clinical trial design, we have no ability to measure biological activity outside the site visit. In late phase trials, site visits are greatly diminished and opportunities to collect biological samples are minimal. Remote sampling or mobile phlebotomy could be very powerful for pharmacokinetics, pharmacodynamics or safety monitoring in this paradigm [37,38]. Remote collection of samples for viral shedding could be powerful for vaccine development. Episodic diseases with acute treatment could benefit from the ability to sample at the time of an event. High risk, vulnerable populations could collect samples at home without the burden of travel and potential exposure risk [31]. In addition, these approaches may enable longitudinal sampling in new ways. This provides opportunities for generating real world data enabling a better understanding of health and disease ‘in the wild’. It can also provide critical data for earlier intervention.

We can use technology to build more complete data sets while minimizing participant burden with less travel and time away from work, school and family. Remote sampling capability has great potential [35,38]. However, there is risk associated with these approaches. If a collection is self-collected and unobserved by site staff, sample chain of custody, sample quality and sample integrity must be addressed. For remote phlebotomy, risks associated with chain of custody are lower. The bioanalytical scientist may need to wear several hats – assisting in site and participant training, developing sample quality checks and conducting additional validation assessments to understand sample integrity and stability in the remote collection paradigm. All stakeholders must collaborate to successfully implement alternative approaches in clinical research.

## Matrix selection

To date the clinical trial paradigm, and even the entire healthcare system relies heavily on data generated from a traditional venipuncture sample. By default, we then isolate plasma or serum from collected blood because this is what has been done historically. Assays are built and many routinely analyzed safety panels are well established in these matrices. However, the pandemic provided an opening to conduct sample collection and analysis in a different way. There may be specific research areas, such as antigen shedding in the vaccine space, where noninvasive sampling or remote collection could provide critical data in clinical trials [31,35]. There are many analytes that can be measured in alternative matrices – dried blood spot (DBS), milk, urine, saliva, skin, breath condensate and hair. In addition, samples can be collected as liquid or dried on different sorbents. Applications for DBS are widespread in healthcare and have been successfully applied to multiple endogenous analytes including universal newborn screening. Small molecules, large molecules and nucleic acids have been analyzed using DBS for genomic, transcriptomic, proteomic and metabolomic markers [36]. The inclusion of the DBS in the FDA bioanlaytical method validation guidance document in May 2019 demonstrates uptake of DBS for pharmacokinetic analysis in the industry [39]. In recent years, we have seen the development of novel polymer-based drying sorbents that resolve hematocrit issues [40]. There are also new ways to collect capillary blood beyond fingerstick sampling, which could simplify collection and decrease pain [41–43].

Bioanalytical assays in different matrices will need assay development and validation that ensures adequate accuracy, precision, stability, etc. These matrices may need more method development as matrices or sample types may be novel. Extraction techniques may need optimized especially in samples in the dried state. In addition, validation workstreams may need adjusted [44,45]. What sample stability needs established for samples stored at room temperature? How do we mitigate issues of sample homogeneity? These, and others, are important questions to be answered in the service of advancing these emerging capabilities.

In cases where nontraditional matrices are incorporated in development programs for decisional purposes, sponsors should engage with regulators to gain consensus on the intended use of the data. In some cases, alternative matrices will need bridged with traditional approaches. For example, pharmacokinetic measurements in dried blood often need bridged with historical plasma datasets [46,47].

The analytical community should think strategically about the biological matrix and determine if we are selecting matrices based on optimal science and patient burden or on inertia. We tend to be risk averse in healthcare and our workflows tend to be over reliant on historical precedent.

## Sample volume

In addition, the healthcare community should consider decreasing the volumes of collection. Technology has evolved such that the sample volume needs in analytical assays are smaller and smaller. Detection limits are continually improving. However, a corresponding drop in the amount of blood collected from patients has not occurred. In most cases, milliliters of blood get drawn and only a small fraction of this volume is utilized for analysis. This creates complexity and burden on the entire process, which is rapidly becoming less and less necessary [48,49]. There are also populations where this excess volume is not just wasteful but can be harmful to very sick individuals [50]. Scientists and instrument manufacturers should continue to increase assay sensitivity, and patients should realize the benefit of decreased collection volumes. Efforts at sample minimization are often referred to as micro sampling. Micro sampling has gained widespread application in preclinical studies in the pharmaceutical industry. A survey of the pharmaceutical industry reported that micro sampling was being implemented in preclinical studies to reduce the number of animals and enable serial sampling. However, uptake in the clinical space has been slower [51]. Sponsors should leverage the learnings from establishing micro sampling in the preclinical space to implement in clinical trials as well. In addition, many novel sampling devices collect smaller volumes of capillary blood or other matrices. Developing assays with smaller volume needs will enable new technology applications with devices that may be less invasive and less painful [42,44,47].

## Device development

Manufacturers of alternative devices have found themselves overwhelmed with demand during the pandemic. The need for new collection approaches has been intense during the last year. Moving forward, we hope this spurs additional investment and corresponding development of new devices for biological sample collection. The vacutainer was invented in 1947 [52]. While it works reliably, there are many instances when alternative collections can provide important data for drug development and decrease patient burden. We should use technology to develop patient centric sampling that is less invasive, less painful and more convenient for patients. These approaches can be combined with decentralized trials to enable innovative virtual trials [35,53,54]. The bioanalytical community will need to develop and validate assays that enable remote sample collection. As data quality cannot be compromised, the data generators are a key stakeholder in establishing these capabilities.

In addition, the bioanalytical scientist should be influencing patient centric device development. Ensuring that manufacturers understand the needs and the downstream workflow will increase the probability of success. In the early phase of device development, it is helpful to build relationships with manufacturers to inform device advancement. Laboratory scientists may need to conduct device testing to assess performance, understand sample quality, develop assays, optimize collection instructions and establish downstream operational testing workflows [47,49]. Although this can be seen as an extra burden on the bioanalytical laboratory, it fosters successful technology development, provides unique development opportunities for innovative thinkers and ensures manufacturers are thinking about the analytical laboratory. Samples have no value if bioanalysis is not possible due to issues with getting a sample out of a device and into the appropriate tube or plate for subsequent analysis.

Alternative sampling approaches can result in new sample types that can be disruptive to existing laboratory processes. Operational capability must be built in the laboratory to handle analysis. This can provide opportunities for novel sample handling techniques and automation options. Scalability of sample analysis can be a concern with incorporation into large, late phase trials. Laboratories must have appropriate automation and throughput capability to manage sample storage and analysis.

## Modernizing the bioanalytical laboratory

In addition, to the changes in clinical trial conduct and sampling, COVID-19 also provided impetus for change in the operation of the bioanalytical laboratory [31]. With lockdowns and travel restrictions, testing facilities were faced with unprecedented challenges. Entirely new ways of working have been developed as scientists moved to virtual platforms whenever possible to enable working from home [35,55]. Remote operation of instruments, electronic document signature, increased automation and virtual meetings were increasingly utilized to reduce the number of people in the laboratories. Virtual audits have become commonplace [55]. The balance of remote and in person work may shift in the coming years as companies and researchers find an equilibrium that optimizes productivity, scientific achievement and work/life balance.

## Conclusion

A theme throughout every area of our lives in the last year has been increased, use of technology to develop new ways of working, connecting and collaborating. A bright spot in all of this is the opportunity to rethink how we leverage technology to make our lives better from the bench scientist, to the patient, to the researchers working so hard to find better treatment for human disease.

In the future, clinical trials will be more patient centric. Clinical site visits will be diminished and more clinical trial conduct will migrate into participants’ homes. We are only beginning to see this transition which has accelerated during the pandemic. Decentralized trials through the use of technology will provide access to clinical research on an unprecedented scale. As long as individuals have access to the internet, they can enroll in trials without needing to be geographically co-located. If applied correctly, this paradigm shift can increase trial diversity, better assist underserved populations and improve treatment access globally.

As the industry moves toward more patient centric trial design, the need for novel sample collection techniques will increase. The future of sampling will not be dominated by the traditional vacutainer. Sampling will not be confined to liquid blood collection. Collection volumes will diminish, sampling will become less painful and samples will be collected outside the traditional site visit. It behooves the bioanalytical community to be prepared for this evolution. Laboratories that do not want to be left behind should invest in developing expertise in new matrices, different sorbents, smaller sample volumes and novel devices. In addition, laboratories should develop scalability plans to ensure they can handle new sample formats at scale.

We are all involved in clinical research because we want patients to thrive. We are obligated to consider the entire journey of trial participants. Where can we leverage technology to decrease patient burden? How can we get more data out of trials to understand biology better?

## Future perspective

Technology has existed for decades to decentralize clinical trials. The pandemic is creating a permanent shift in clinical trial conduct. Sponsors and investigators are overcoming the administrative and bureaucratic hurdles to provide more patient centric clinical trials. Incorporating novel technology for enrollment and trial conduct will become the new norm. Patient centric clinical trial design will increase enrollment and retention in clinical trials by decreasing patient burden. In future, these approaches will improve treatment access and clinical trial diversity as trial participants will not need to be geographically co-located with clinical sites. Patient centric sampling will enable decentralized trials and provide opportunities to build more robust datasets during drug development. In addition, alternative sampling approaches will support efforts to enhance real world evidence in understanding health and disease. To meet ever increasing demand for alternative sample collection, manufacturers will continue to build novel devices that decrease sample collection volumes, provide painless collection and enable remote sample collection.

**Executive summary**

- Adoption of new technology to increase clinical trial conduct efficiency and create a more patient centric paradigm has been slow in the pharmaceutical industry.
- The pandemic has had a huge impact on clinical research – stopping over 2000 clinical trials. Out of necessity for maintaining social distancing during COVID-19, sponsors have built capability around virtual, decentralized clinical trial approaches that are inherently more patient centric.
- Collection of biological samples outside of traditional clinical site visits has gained importance in light of COVID-19 lockdowns and travel restrictions. Novel collection techniques provide decreased collection volumes, decreased pain and opportunities to obtain samples remotely.
- Novel collection approaches are an enabling technology for decentralized, patient-centric trial design.
- The bioanalytical community must build processes to support patient centric sampling by building assays, exploring new matrices, collaborating with manufacturers on device design and establishing new operational workflows.
